# Maternal Healthcare Provider Perspectives on Spiritual Care and the Challenges and Opportunities for Provider Spiritual Well-Being in the United States

**DOI:** 10.1007/s10943-025-02515-z

**Published:** 2026-01-08

**Authors:** Tyler S. Nesbit, Karen A. A. R. Coker, Michelle Abraczinskas, LaToya J. O’Neal, Sabine Grunwald, Sarah L. McKune, Larry F. Forthun

**Affiliations:** 1 Department of Family, Youth and Community Sciences, College of Agricultural and Life Sciences, University of Florida, Gainesville, FL USA; 2 Global Pediatrics Program, College of Medicine, University of Minnesota, Minneapolis, MN USA; 3 Department of Soil, Water, and Ecosystem Sciences, College of Agricultural and Life Sciences and UF Mindfulness Program, University of Florida, Gainesville, FL USA; 4 Department of Environmental and Global Health, College of Public Health and Health Professions, University of Florida, Gainesville, FL USA

**Keywords:** Spiritual care, Maternal health, Interview study, Qualitative analysis, Thematic analysis

## Abstract

Spiritual well-being is essential to overall well-being for healthcare providers. Furthermore, providing spiritual care depends on providers’ spiritual well-being and competence in assessing patients’ spiritual care needs. Including spiritual care in maternal healthcare represents an opportunity to improve the quality of patient care. We conducted interviews with 20 maternal healthcare providers to understand how to address spiritual care implementation goals. Through thematic analysis, the following themes were identified: defining spirituality, the interplay of provider spirituality and spiritual care, and the barriers and facilitators to support provider well-being. Opportunities to support provider well-being, an essential piece of developing spiritual care programs, are discussed.

## Introduction

Healthcare providers in the USA are facing their own health crisis, resulting in high rates of moral distress, compassion fatigue, and burnout (U.S. Surgeon General, 2022). The Covid-19 pandemic both exacerbated and highlighted the need to address healthcare providers’ well-being (Lluch et al., 2022). Maintaining a high level of provider well-being is essential for the healthcare system to provide high-quality care for patients (Salyers et al., 2017). Because the causes of burnout and moral injury among healthcare providers span across individual (e.g., physical and psychological distress), organizational (e.g., administrative demands), and social (e.g., workforce shortages) levels, a single approach to address this issue is unlikely to be effective (Sipos et al., 2024). Rather, it is necessary to develop a suite of multilevel approaches that support providers in multifaceted ways. This study focuses on spiritual well-being as an element of overall provider well-being.

In healthcare, spirituality has been defined as “the way individuals seek … meaning and … experience their connectedness to the moment, to self, to others, to nature, and to the significant or sacred” (Puchalski et al., 2009, p. 887). Spiritual health is a dimension of overall well-being that deserves attention for its potential to protect against provider distress as well as improve patient care. For example, a review of 25 studies found that provider spirituality served as a method to cope with stressors and protect the mental health of providers, which resulted in greater patient satisfaction with the quality of care (de Diego-Cordero et al., 2022). Holistic patient care includes spiritual care, an approach that attends to a patient’s sense of meaning, hope, and inner strength and prioritizes patient wholeness and healing over treating a specific condition or disease (Kelly, 2012; Puchalski, 2001).

Spiritual care interventions have been found to improve patient spirituality, well-being, mental health, pain management, and biophysical measures of heart rate, blood pressure, oxygen saturation, as well as motivation to engage in health-related behaviors (Anderson & Pullen, 2013; de Deigo-Cordero et al., 2022; Guilherme et al., 2016; Puchalski et al., 2018). Additional research indicates that a provider’s own sense of spirituality and their spiritual well-being are central to the successful implementation of spiritual care best practices for patients (Jones et al., 2021; Paal et al., 2015). Therefore, provider spiritual well-being is essential to provide high quality, whole-person care, in general, and spiritual care, in particular.

Certain areas of medicine are more likely to see an immediate benefit from a focus on the spiritual well-being of providers and patient spiritual care. For example, the current applications of spirituality in healthcare predominantly focus on the palliative care setting, where spiritual care has been established as a core competency to aid patients in facing existential questions related to end-of-life care (Miller et al., 2023; Puchalski et al., 2009, 2022). Another area of medicine that presents opportunities for the application of spiritual care is maternal healthcare, the care for a patient during pregnancy, childbirth, and the postnatal period (World Health Organization, 2025). The spiritual dimension of childbirth is significant for expectant parents (Crowther & Hall, 2015), and maternal healthcare providers and researchers have identified attention to spiritual well-being as an important aspect of improving care. For example, the Cycle to Respectful Care framework calls for providers to “create an environment where patients feel secure and supported in their cultural, spiritual, and religious practices” (Green et al., 2021, p. 7).

Spirituality has been shown to play a key role in the process of making meaning of major life transitions, such as the transition to parenthood (Grazina et al., 2025; Redelinghuys et al., 2014). Furthermore, there is good reason to anticipate that routinely incorporating spiritual care into maternal healthcare will improve outcomes for patients, responding to calls to increase access to high-quality maternal healthcare from preconception through the post-partum period (Ahn et al., 2020; Nichols & Cohen, 2021). Research has found positive associations of spiritual care with health outcomes of women with preeclampsia, gestational diabetes, stress, anxiety, depression, and quality of life (e.g., Kamali et al., 2018; Sanaeinasab et al., 2021; Xu et al., 2025). A randomized controlled trial among women experiencing anxiety during preterm labor found that patients who received a spiritual care intervention reported significantly lower levels of anxiety than control group participants (Maasoumi et al., 2023). Another qualitative study reported that spiritual care and empathy improved women’s birthing experiences and this carried over into improved postnatal care (Moloney & Gair, 2015). Despite this accumulation of evidence, and its integration in palliative and end-of-life care (e.g., Puchalski et al., 2022), spiritual care remains elusive for beginning-of-life perinatal care (Crowther et al., 2021).

A systematic review of existing programs to train providers in spiritual care related to childbirth identified only two studies, both from a single project (Prinds et al., 2021). One reason for a shortage of training opportunities for spiritual care may be the need to understand providers’ perspectives and attitudes toward such interventions. In practice, provider views of spirituality are not necessarily the same as patient views. For example, Selby reports on the similarities and distinctions of spirituality from the perspectives of patients and providers and how the differences can interfere with providers’ attempts to administer spiritual care, resulting in them missing opportunities to identify and address issues related to patient spiritual well-being (Selby et al., 2017). Research on patients’ perspectives of spirituality in healthcare indicates patients’ willingness for providers to engage with spirituality and spiritual care in general (Best et al., 2016), and within maternal care (Backes et al., 2022). However, no studies have focused on providers’ perceptions of spirituality and spiritual well-being in the US maternal healthcare system.

In recognition of the potential benefits of integrating spiritual care into maternal healthcare, and the need to develop spiritual care programs within the maternal healthcare context, this study focuses on the perceptions of maternal healthcare providers related to spirituality and their own spiritual well-being. Attention to providers’ perspectives also prioritizes implementation targets such as acceptability, appropriateness, and feasibility by ensuring that the needs of healthcare providers are understood and addressed in intervention design (Proctor et al., 2011).

### Purpose of the Current Study

This study seeks to understand providers’ views as a necessary step in developing spiritual care training programs in maternal healthcare, which benefit providers and patients. The objectives of this study are to examine maternal healthcare providers’ experiences with spirituality and identify facilitators and barriers to maternal healthcare providers’ spiritual well-being.

## Methods

This study used a qualitative methodology guided by interpretivism to conduct a series of in-depth, semi-structured interviews. All methods and results are reported according to the consolidated criteria for reporting qualitative research (COREQ) (Tong et al., 2007).

### Participant Selection

Eligible participants were defined as physicians, nurse practitioners, and nurse midwives specializing in primary care, obstetrics, labor and delivery, and post-partum care. Participants were purposefully sampled based on their level of experience as providers, their institutional affiliation, and their demographic characteristics, including race, gender, religion, and spiritual orientation. Participants were approached via email using their institutional email addresses. A sampling quota was used to ensure a diverse sample. According to Robinson (2014), quota sampling is a flexible purposive sampling strategy that ensures the inclusion of key groups in the sample. According to research indicating differences in attitudes, knowledge, and skills related to spiritual care, participant gender, race, and religious identity were deemed to be key attributes related to the research questions (Dillard et al., 2021; Smyre et al., 2018). Therefore, a quota sampling strategy was used that included a minimum of four male participants, four African American or Black participants, and four nonreligious participants.

The concept of information power (Malterud et al., 2016) was used to guide the sample size, which included the study aim, sample specificity, use of theory, quality of dialogue, and analysis strategy (Malterud et al., 2016). Considering all five items for this study, the expected information power from each interview was expected to be moderate, and a medium-sized sample was expected to be sufficient. A review of six qualitative studies with providers reporting on spirituality in healthcare reveals sample sizes ranging from 8 to 23 participants (Best et al., 2016; Considine, 2007; de Diego-Cordero, 2023; Rajabipoor & Mohammadi, 2021; Rogers et al., 2020; and Selby et al., 2017). Based on this preliminary analysis of information power and the typical sample sizes for similar studies, a sample size of *n* = 16 participants was proposed. The information power of interviews was continually assessed as data were collected and a final sample of 20 was determined based on this reflexive assessment. No participants refused to participate or dropped out.

### Ethical Considerations

Although the study was determined to be exempt from IRB review by the University of Florida IRB office, participants were provided with an electronic copy of an informed consent document detailing the purpose of the research, as well as any potential risks and benefits of participation. Informed consent documents were provided in advance of data collection so that participants had time to review on their own time frame. Data, including transcribed audio and video recordings, were de-identified to protect participant privacy. Participants were offered $50 Amazon e-gift cards in appreciation of their contribution to the study.

### Data Collection

Interviews were conducted by a member of the study team (TN), a doctoral candidate and graduate research assistant trained in qualitative research. He had no prior relationship with the participants, and he introduced his personal and professional interests in the research prior to each interview; namely, as a spiritually oriented, White man married to a Black woman with prior personal experience in maternal healthcare. An interview guide including a set of questions and follow-up probes was developed and used to initiate dialogue with participants (see Appendix 1). Questions were developed based on preliminary research in the study area and the guiding research questions for the first author’s dissertation study. The guide was pilot tested to improve the wording and order of questions. For example, questions included, “Was there ever a time when you felt that your sense of spirituality contributed to your career choice as a maternal healthcare provider? Can you tell me about that?” and “Where do you see opportunities to support the spiritual well-being of maternal care providers in the delivery of your medical care services?” In-depth interviews were focused to ask questions in a way that evoke deep reflection and sharing by the participant. Interviews were conducted online (via Zoom) and in person, depending on the participant’s availability.

For in-person interviews, a suitable location with minimal distraction was selected. Audio was recorded using a digital recording device, and notes were taken by hand on a printed version of the interview guide with ample space for notetaking. For in-person interviews, an iPhone setup on a tripod was used to video record the participant to capture the participant’s facial expressions and body language. On Zoom, participants were requested to keep the video option on and to be in a place where they could meet uninterrupted for the duration of the interview. The audio and video were recorded using Zoom’s built-in recording feature. Audio files from face-to-face interviews were uploaded from the recording device to the computer, and Zoom interview files were downloaded from the Zoom platform. Recordings were uploaded to the Otter.ai platform for transcription. These initial transcripts were uploaded into the qualitative data analysis software ATLAS.ti for review. The lead author reviewed each transcript while watching and listening to the audiovisual interview recordings and edited for accuracy. Transcripts were provided to participants for review prior to analysis.

### Data Analysis

This study used inductive thematic analysis of transcribed interview data to answer the research questions based on general approaches to qualitative inquiry described by Braun and Clarke (2006), Mihas (2023), and Saldaña (2016). Themes were discerned from the collected data, which explore how participants understand spirituality and integrate practices to foster their own spiritual well-being and that of their patients. The procedural stages of data collection and analysis temporally overlapped. Data analysis began with listening and viewing audio and video recordings of the interview, transcribing the audio recordings, and memo writing.

Memos were employed throughout as a way for the research team to familiarize themselves with the data and to create “empirical intimacy” (Truzzi, 1974). Multiple memo types were employed including key quotation memos, document reflection memos, positionality, and comparison memos. Memo writing and coding continued iteratively to reveal the “thickness” of the data (Terrell, 2016, p. 166). This process continued for each interview while continuing to conduct additional participant interviews. This ongoing process of data collection and analysis helped to reflexively adjust interview questions, assess the informational power of the sample, and determine the study sample size. Like memo writing, the coding process was iterative. The data contained in the form of the transcripts were inductively coded in multiple passes of analysis and interpretation of the data for patterns within and across participant interviews. Initial coding of the phase one interview transcripts used an eclectic approach including In-Vivo Codes, Descriptive Codes, Process Codes, Values Codes, Attribute Codes, and Structural Codes (Saldaña, 2016).

Following the initial coding of transcripts, peer debriefers were recruited. The study benefitted from three rounds of peer debriefing with two postdoctoral qualitative researchers (April 18, April 26, and May 24, 2024). The goal of the peer debriefing process was to enhance the credibility of the analytic process (Terrell, 2016). In the second cycle of coding, a round of structural coding was conducted to organize the data and focus the analysis on addressing the research questions. Coded quotations were compiled and used to generate new text documents that included similar content across participants.

The documents were coded for initial themes. Resulting codes were then compiled into an Excel spreadsheet and sorted alphabetically. They were then rearranged and grouped with similar codes to identify themes and subthemes. Themes were described along with reflections on the process in a prose format interspersed with representative sample quotations from interview transcripts. A brief report on the themes was submitted to participants for member checking, a process which enhanced the credibility of the findings, confirming internal validity.

## Findings

### Overview of Study Participants

All interviews were audio recorded and, except for one participant, video recorded. Interviews were conducted in person (*n* = 5) and via Zoom (*n* = 15). The duration of interview recordings ranged from 55 to 101 min, with an average of 79 min. In addition to the attributes identified in the quota-based sampling approach (gender, race, and religiosity), the sample was also diverse according to provider role, training, specialization, and years of experience (see Table 1). Most of the participants identified as women (70%), with 20% men, and 10% nonbinary gender participants. Participants were all employed as maternal healthcare providers at the time of the interviews across a total of six institutions in three states.

**Table 1:** Participant characteristics

Provider Role	*n* (%)*	Experience	*n* (%)	Race	*n* (%)	Religion	*n* (%)
Medical doctor (MD)	9 (45)	10 +	8 (40)	White, non-Hispanic	10 (50)	Spiritual**	17 (85)
Midwife (LM, CNM)	8 (40)	5–10 yrs	5 (25)	White, Hispanic	3 (15)	Christian	11 (55)
Nurse practitioner (NP)	4 (20)	2–5 yrs	4 (20)	Black and African-American	5 (25)	N/A	7 (35)
Physician assistant (PA)	1 (5)	1–2 yrs	2 (10)	Middle Eastern	1 (5)	Muslim	1 (5)
Registered nurse (RN)	1 (5)	< 1 year	1 (5)	South Asian	1 (5)	Pagan	1 (5)

^*^These percentages sum to greater than 100% because some providers hold multiple credentials, e.g., CNM and NP

^**^Spiritual participants include both spiritual AND religious and spiritual NOT religious

Themes were organized into three domains: defining spirituality, provider well-being and patient spiritual care, and facilitators and barriers to supporting providers’ own spiritual well-being (see Table 2). Findings are presented below by domain.

**Table 2:** Organization of findings by domains, themes, and subthemes

Domains	Themes	Subthemes
Defining spirituality	1) Spirituality is a way of defining purpose and finding meaning	1a) Spirituality is related to religion and belief in a higher power
		1b) Spirituality offers a way of understanding one’s place in the world
		1c) Spirituality is metaphysical
	2) Spirituality is a personal experience	
	3) Spirituality is about connection	
Provider well-being and patient spiritual care	4) Provider spiritual well-being is favorable for providing spiritual care	
	5) Spiritual care involves navigating different beliefs of patients and providers	
	6) Provider spirituality is affected over time through providing maternal healthcare	
Facilitators and barriers to supporting providers’ own spiritual well-being	7) Individual-level facilitators	7a) Skills and behaviors in the clinic
		7b) Self-care behaviors outside the clinic
		7c) Personal attributes
	8) Individual-level barriers	8a) Burnout
		8b) Isolation
	9) Organizational-level facilitators	9a) Structural support
		9b) Cultural support
	10) Organizational-level barriers	10a) Competing institutional priorities
		10b) Malignant work culture
		10c) Societal issues

### Defining Spirituality

The themes in this section respond to the goal of understanding how maternal healthcare providers define spirituality. Providers variably described spirituality in terms of recognizing and relating to a higher power, a way of making sense of the world and one's role in it, a sense of connection, a process of inquiry, mysterious and unknowable, as culturally situated, informal, personal, and fluid over time. However, three primary themes were identified: 1) spirituality is a way of defining purpose and finding meaning, 2) spirituality is a personal experience, and 3) spirituality is about connection. Below is an in-depth description of each of these themes, subthemes, and exemplar quotations.

#### Spirituality is a Way of Defining Purpose and Finding Meaning

This theme was identified as a broad description of the three subthemes presented below, which are that spirituality is defined through religion and belief in God or a higher power, that spirituality is a way of understanding one’s place in the world, and the view of spirit as distinct from mind and body. These three subthemes are connected by the overarching theme of understanding spirituality as a way of defining purpose and finding meaning.

**1a. Spirituality is related to religion and belief in a higher power.** When asked about spirituality, providers commonly described their religious orientation. Even those who did not identify as religious frequently referenced their religious upbringing. Perspectives on this relationship varied, with some describing spirituality and religion in terms that implied equivalence and most considering spirituality as a broader umbrella term that can, but does not always, include religion. The idea of spirituality as having to do with recognizing and relating to a higher power was commonly expressed. One participant expressed this view in the following way, “ultimately, I … recognize that there’s a higher power, that there’s an order to things” (P18, NP, CNM, Christian).

**1b. Spirituality offers a way of understanding one’s place in the world.** The idea of spirituality as a way of making sense of life, either on a grand scale or in one's personal life experiences, was identified as a subtheme. “Spirituality is kind of how you fit into the greater scheme of the world” (P09, MD, Muslim). Related to this idea of our place in the world is the perspective that spirituality offers a way to guide our actions, which includes a passive aspect, “[spirituality] gives you a foundation and gives you some guidance, some guidelines, on how to live your life” (P03, NP, Christian) and an active aspect, “there's something that's higher than myself, that helps me to kind of go about life. And I feel like there's something higher than me that helps govern my life” (P07, LM, Christian). These quotes demonstrate how spirituality can provide a framework to interpret one’s role and relationship to the world, and ways in which individuals can access guidance by referring to the roadmap laid out by their spirituality or through actively seeking guidance from a higher power.

**1c. Spirituality is metaphysical.** This subtheme speaks to the view that spirituality has to do with spirit, a category distinct from the physical, and is also related to finding purpose and meaning. This perspective posits that spirit is an essential, or the essential, component of human existence and that spirituality is based on this awareness. This view was expressed by a participant, “spirituality is acknowledging that there's a component of yourself outside of the physical … that we're more than mind–body, but we're mind, we're body, we're spirit” (P08, PA, Christian). Another participant related this ontological perspective to the overarching theme of spirituality as a way of finding purpose and meaning, “the practice of spirituality is the recognition that one is a spirit and … orienting their life around that truth” (P17, CNM, Christian).

#### Spirituality is a Personal Experience

The second theme related to defining spirituality was identified as spirituality being a personal experience. The idea of spirituality as personal was discussed in terms of the informal nature of spirituality, a contrast with the formality of religion. Additionally, practicing spirituality was described as a process of introspection and self-inquiry, which leads to an evolving understanding over time. In other words, engaging with spirituality involves a lifelong personal journey. One participant stated:

Spirituality is to be in touch with your inner self, which I believe we call spirit, and ask the question, what is the meaning of this universe or what's happening around us? … I think that constant asking is integral to spirituality, because as long as you exist in this universe … the questions never end. And actually, the longer you live, you end up with more questions. (P02, MD, spiritual not religious)

Spirituality was further described in various emotionally descriptive and experiential terms. For example, one provider said, “the moments when I have felt spiritual, you know, or felt spirit, the feeling that I receive is like a deep introspection… Something that was felt with … intense emotion that was tied to that particular event” (P13, CNM, not spiritual nor religious). These ways of understanding spirituality as personal and experiential were united under these personal lived experiences.

#### Spirituality is About Connection

The final theme in the domain of defining spirituality relates primarily to connection with other people. The focus was on a shared experience of community and friendship, maintaining connection to loved ones, living and deceased, as well as a sense of being connected to one’s own being. Interestingly, this theme was predominant among participants that identified as nonreligious. However, there is room to include the natural world, connection to self, and connection to God or a higher power as well. One provider explained:

We're all doing the best we can. We're all born as … these innocent little beans that have no idea what's going on. And we're just here to help each other. We're all in the same boat…When I think about being spiritual … I think love, friendship, connecting with other people and understanding that we're all made of the same thing. (P15, NP, CNM, spiritual not religious)

The themes and subthemes described above articulate the essential aspects of how the maternal health providers in this study conceptualized and defined spirituality. The next section details themes related to the enactment of provider spiritual well-being into practice for patient spiritual care.

### Provider Spiritual Well-Being and Patient Spiritual Care

The following three themes emerged relating to how providers view the relationship of their own spiritual well-being to the practice of implementing spiritual care for patients: provider spiritual well-being is favorable for providing spiritual care, providing spiritual care involves navigating differences between patients and providers, and provider spirituality is affected over time through providing maternal healthcare. Each theme is described below with supporting quotations; no subthemes emerged during coding.

#### Provider Spiritual Well-Being is Favorable for Providing Spiritual Care

Participants generally held favorable attitudes about the potential for spiritual care as a positive contribution to maternal healthcare, as well as the relationship of provider spiritual well-being to providing spiritual care. The bidirectional impact of provider spiritual well-being and providing spiritual care was addressed across interviews. “Nursing overall, I think, was part of my spirituality, of caring for other people, showing compassion and showing empathy towards someone who needs your professional care” (P20, RN, Christian). The idea of empathy was recognized as an aspect of providing spiritual care that enhanced the spiritual well-being of providers and was bolstered by having traversed challenging life experiences. For example, one participant shared, “unless we suffer from some personal tragedies, we don't necessarily connect with our spirituality, but I definitely think that you become a much better physician if you are a seasoned patient” (P02, MD, spiritual not religious). The same participant connected their spiritual growth through personal experiences as a patient to compassion and empathy:

I think compassion always exists. You know, because when I make recommendations based on the strength of scientific evidence, I'm doing this out of compassion … empathy is to understand what is the endpoint that patient wants to go, and how she wants to go there. (P02, MD, spiritual not religious)

Empathy as a maternal healthcare provider was acknowledged as being helpful for patients to have a positive birth experience, “it's their one experience. This is the one time they're having the baby, even though you see this every day” (P17, CNM, Christian). Providing spiritual care also meets a spiritual need for many providers who see their work as a calling:

My spirituality, I think it's an awareness of that I'm more than one part and the people I care [for], you know, one of the things that I do is I look at what I do as a calling. I couldn't do my job if it weren't a calling. (P08, PA, Christian)

#### Spiritual Care Involves Navigating Different Beliefs of Patients and Providers

While valuing the role of spiritual care for patients and provider well-being, providers also described the complications to consider with respect to providing spiritual care for patients with whom they do not share beliefs. “I mean, it's interesting to have your own kind of strong spiritual or religious beliefs, and then also try to have the awareness that like, other people might not hold those” (P17, CNM, Christian). Maintaining professionalism was acknowledged as imperative to being a good provider, and the extent to which providers forefront their own spirituality was described as being in tension with this goal at times. One provider offered, “I don't want to offend anyone. And I also … feel people, some people really do have a prejudice or a bias against … Christian people, or even people who practice religion” (P18, NP, CNM, Christian). Another said, “the beliefs that … I've grown up with, aren't necessarily the same thing that my patient has. So, I have to really kind of compartmentalize those things” (P03, NP Christian). It is important to note that not all providers struggled with navigating differences in spiritual or religious belief with patients. Although acknowledging and navigating differences in belief with both patients and colleagues was a theme that was identified with respect to providing spiritual care, some providers were clear about the appropriate boundaries.

#### Provider Spirituality is Affected Over Time Through Providing Maternal Healthcare

In addition to negotiating spiritual care across differences between patients and providers, some expressed how their own spiritual perspective had changed over time, resulting from their work in maternal healthcare. Providers acknowledged that their experiences in the field had affected their understanding and practical application of spirituality. One indicated how their appreciation for the positive role of spirituality in the lives of their patients and colleagues had grown over time despite their own initial aversion to spirituality:

Spirituality was something I really pushed away. And I almost found myself having trouble understanding people when that was very important to them … still, I don't identify as a spiritual person. But I definitely respect people who are a lot more than I did … Especially as I've seen people go through very difficult life events. It's a lot easier to understand the importance to people when you're seeing them in some of their hardest times, in … what they think are their worst versions of themselves. And that's patients or coworkers. (P04, MD, not spiritual nor religious)

Spirituality was most often discussed in terms of growing or deepening over time through witnessing the process of childbirth and patients responding to adversity. Regarding childbirth, one provider said, “when you think about the science behind having a baby. It's like, all the stars have to align for it to happen. This thing goes from one little cell to actual human being, it's amazing” (P03, NP, Christian). However, providers discussed how spiritual well-being was challenged at times, due to strong feelings of sadness or frustration associated with adverse events despite the best medical attention and care, or due to the broader sociopolitical context. For example, one provider recounted, “It took me one year or more to get over the death of that baby… the thought of somebody leaving the hospital without a baby they were expecting tears me apart” (P19, MD, spiritual not religious). The context of intersecting religious organizations and healthcare politics also challenged some providers conception of spirituality and the relationship to religion:

So, the sense of spirituality, I don't think that has changed much. That remains. But my personal ability to go down the religious pathway, … that's become much more difficult. …I mean, we do women's health, there's a lot of gray area. And religion wants there to be a lot of black and white, and we constantly sit in that in-between. And so, I see the limitations that religion puts on my ability to care for women. And that makes it very difficult to say, yes, I can believe in that. (P14, MD, Christian)

These examples illustrate how participants integrate their experiences as maternal healthcare providers. The process of intimately observing and participating in patient experiences that are spiritually and emotionally laden often connect to a providers’ own sense of spirituality. These experiences impact the spiritual well-being of providers and, as identified in theme 4 (provider spiritual well-being is favorable for providing spiritual care), their ability to provide spiritual care for patients.

### Facilitators and Barriers of Maternal Healthcare Providers’ Own Spiritual Well-being

Generally, facilitators and barriers for provider spiritual well-being were discussed at the levels of the individual and the organization (see Table 2), which served as the themes for this domain. For each theme, the subthemes and example quotations are provided below.

#### Individual-Level Facilitators

At the individual level, three facilitators of providers’ own spiritual well-being were identified as subthemes: skills and behaviors at work, self-care behaviors outside work, and personal attributes that protect well-being at work.

**7a. Skills and behaviors in the clinic.** Providers described an assortment of learned skills and behaviors to promote spiritual well-being. Providers expressed the importance of being prepared for patient visits by reviewing charts and previous communication:

I like to be very prepared. I think that's the biggest thing I do to support myself is I prep my charts every day. So, the day before I write out all my patients and like, what I've seen them for, you know, questions that I want to ask them what I think the visit is going to be about based on what their primary complaint is. So, I feel like that's the biggest sign of respect is to go in prepared. (P15, NP, CNM, spiritual not religious)

When patient encounters become strained, helpful skills include taking deep breaths, avoiding ruminating on negative experiences, and reframing difficult encounters with patients. For example, one participant shared, “when you … get upset because a patient's rude or angry… They're not angry at us. That's really egotistical. They're angry at the situation” (P05, DNP, CNM, not spiritual nor religious).

Providers also highlighted the role of receiving social support from other providers:

The people that I have around me are just … very good on picking up our emotions and like, what we're going through and what we're feeling, because she noticed it immediately and she was like, hey, everything's fine. Like, take a breath. Over the last month, she multiple times reminded me, just take a breath, everything's okay, everything's going fine. But I think having each other to look out for one another is really helpful. (P11, MD, spiritual not religious)

Participants emphasized setting boundaries to protect time off, including breaks during work hours. In describing the benefits of setting boundaries, a participant said:

I think they are 100% better providers because they're healthier. I mean, it's, just like as we tell moms like, you have to take care of yourself, so you can take care of your children. And everyone always uses the airplane, you know, put your mask on first so you can breathe, so you can help the people next to you. It's the same idea. And, you know, I really think that I have no problem saying that without a doubt the people who take care of themselves, who are more emotionally healthy, are better health care providers. (P18, NP, CNM, Christian)

Another participant emphasized the need for providers to take the initiative to set boundaries to protect their time and well-being:

Healthcare is a business. And if you're going to keep going and going, the business is not going to say, “no, no, no, stop.” But I think it's really the individual that needs to say, “hey, I need a time out here.” (P20, RN, Christian)

To review, there are an assortment of skills that providers can develop and practice to protect their own spiritual well-being at the individual level. These include preparation, transparency, perspective taking, connecting with fellow providers in mutual support, and setting strong boundaries. These behaviors are related to spiritual well-being from the perspective of the providers who participated in this study, and fit within the broad scope of the definition of spirituality used for this study, particularly “the way individuals … experience their connectedness to the moment, to self, to others” (Puchalski et al., 2009, p. 887).

**7b. Self-care behaviors outside the clinic.** Outside of the workplace, a wide-ranging list of self-care activities was identified including travel, exercise, time in nature, cooking and eating, taking naps, and entertainment. Directly accessing spiritual resources was also identified, such as engaging in prayer, contemplation, reflection, and practices of gratitude. For example, one participant shared:

I do live by faith. And I know ultimately, no matter what I'm facing, I know God is in control. And I just try to be obedient. And I realize that this world that I live in is going to be full of trials and tribulations. You know, you pick up and you carry your cross. And, and I'm extremely blessed, and I know it. So, I just try to be more thankful than anything. (P06, MD, Christian)

While engaging in prayer for extended periods during the workday is not feasible, providers described taking a moment or two for reflection throughout the day. One described making short, silent appeals to their higher power on behalf of themselves, their colleagues, or their patients as “flare prayers” (P08, PA, Christian). Contemplation and reflection involve the integration of faith traditions into daily life and support providers in transforming the emotional intensity common to their profession into spiritual connection.

**7c. Personal attributes.** Attributes of providers play a role in promoting spiritual well-being. These attributes include lived experiences, personality traits, and an appreciation for the work of maternal healthcare. Participants shared that living through a variety of personal and professional experiences offered an ability to frame the challenges and stressors experienced as a provider. Intrinsic attributes such as provider personality also served as facilitators of spiritual well-being. A participant shared: “I'm the type of person that likes to go, go, go, go go…. I mean, it also goes with my personality” (P01, CNM, Christian). In this case, the participant was describing how the busy work environment was a good fit with their personal preferences, which served as a protection for them against experiencing exhaustion and burnout. An appreciation for the work of providing maternal healthcare was discussed by many participants, which included aspects related to respect for the process of pregnancy and childbirth and a love of providing care for women as exemplified by the following exemplar quotation:

My sense of purpose in my career, has always been to care for women. From the moment I even began nursing school, it's like, this is what I want to do. Especially birthing women. … seeing women at one of the most vulnerable states in their life and empower them through that is very special to me, what women can do and our bodies can do. (P12, CNM, Christian)

#### Individual-Level Barriers

Two barriers to achieving spiritual well-being for providers at the individual level were identified: burnout and isolation. Burnout includes elements of time pressure, exhaustion, and trauma, and isolation involves both personal and professional components.

**8a. Burnout.** The exhaustion of being overworked and stressed erodes a sense of spiritual well-being for providers. Burnout was expressed through words like “exhaustion,” (P08, PA, Christian), “high stress,” (P11, MD, spiritual not religious), and “overworked” (P01, CNM, Christian). A sense of time pressure was frequently mentioned in this regard. “I think the biggest challenge, of course, is time, not taking time away for yourself” (P03, NP, Christian). “Time. Time and resources. We are too thin. We are too thin. We're expected to do too much. Self-care is always on the back” (P08, PA, Christian). “You are limited by time and all the limitations that the system brings. If you want to list every other which thing I feel like so much of it ends up being just time” (P04, MD, not spiritual nor religious). Time pressure may be experienced in relationship to the billable time available per patient visit, the time during a single work shift to see all scheduled patients and complete the corresponding documentation, the time during the day to complete work responsibilities and tend to one’s personal life, including family and personal health, and/or lack of time off for sick days and annual vacation, etc.

The emotional toll of continuously attending to variable and intense emotional moments like birth and death, one patient after another, is a pattern that was prominent across interviews and contributes to provider compassion fatigue:

We close the door to one room, and we drop everything that we just experienced there and walk brand new into something else. And that's just expected of you. You just keep shutting the doors. And that can all be very heavy in the end. (P14, MD, Christian)

Maybe you had a bad outcome, and you haven't had a chance to think about it or talk about it. And then you're expected to go to the next room and deliver another baby or do another C section. (P10, MD, Christian)

The role of provider trauma, including the concept of second victims, was discussed. It was described how providers may “cope” (P05, DNP, CNM, not spiritual nor religious) with alcohol and drugs. These elements of compassion fatigue, secondary trauma, stress, and exhaustion all contribute to provider depersonalization and burnout.

**8b. Isolation.** Providers expressed elements of feeling isolated due to their religion, race, medical specialization, and level of experience. Time pressures and a lack of understanding of the intricacies of medical care by family and loved ones also contribute to feeling isolated:

You know, I go home, and I tried explaining things to my significant other, and he looks at me like I've got three heads, or you're trying to back up and explain, like, what does this mean, and why does that? It's a lot more explanation involved. (P14, MD, Christian)

The stigma of seeking mental health treatment among healthcare workers was discussed:

We would be naive to ignore the mental health crisis amongst physicians and medical professionals because it clearly exists. And a lot of institutions do offer … supports for services … But I think there's a lot of mistrust. And just go to somebody that, you know, you're employed by and try to disclose some of those deeper issues. There's a lot of fear that it could lead to repercussion that you could be looked down upon, you could lose your job that you could lose your licensing. So, I, you know, all throughout my training, it was beaten into us that you do not go to management, you do not go to somebody in your institution, you go far outside of that to seek help. (P14, MD, Christian)

#### Organizational-Level Facilitators

Organizational-level support was indicated as a key element to promoting the spiritual well-being of providers. Facilitators of provider spiritual well-being at this level were identified as related to structures and culture.

**9a. Structural support.** These factors include basic aspects of human resources related to pay, healthcare, childcare, and time off.

Offering fair pay to all parties across the board, offering affordable and high-quality health care that includes both physical and mental health services, offering access to childcare. And then offering I guess, kind of within the institution, a safe space to be able to come and speak needs without the fear of repercussion. (P14, MD, Christian)

Hosting listening sessions for healthcare workers and incorporating formal debriefing sessions, led by trained facilitators, to support provider emotional health following adverse outcomes was suggested.

I think we need to listen to the people that actually work on their feet [gestures for emphasis] on the floor, to the people that provide the care in the clinic and to start solutions from that than just bring in things or doing that or that solution with, when there's no input from the people that actually are doing work, that they're going to tell you exactly what they need in order to provide the care you want. (P01, CNM, Christian)

**9b. Cultural support.** Generally creating a supportive culture that prioritizes personal wellness by promoting taking time off, being with family, and taking care of health was identified as a facilitator for provider well-being.

I'm grateful where I am now. And I would hope that if I go somewhere else, that I would be able to continue it. The providers here of all training levels are all very personally close and personally invested in each other. And it comes it's like a cultural thing that I know isn't everywhere. (P04, MD, not spiritual nor religious)

I try to … encourage people to take time off. Sometimes people will say, “I can't do that, because I have to see patients.” No, the clinic will be okay. Just go take some time… so you got to encourage people to, to take some time off and, and spend time with their family. (P03, NP, Christian)

Positive interpersonal interactions with patients and other providers were also indicated as being supportive of provider spiritual well-being, which may be encouraged through broader cultural initiatives.

#### Organizational-Level Barriers

Three barriers to provider spiritual well-being at the institutional level were identified as subthemes; these were competing institutional priorities, a malignant work culture, and broader societal issues.

**10a. Competing institutional priorities.** Participants described aspects related to institutional priorities that reflect a narrow view of efficiency and short-term profit maximization. This leads to under-staffing, a lack of access to wellness resources for providers, and corresponding time pressures and occupational stressors, which impact the overlapping elements of physical, mental, emotional, and spiritual well-being. For example, the following quotation speaks to the issues that confront providers based on the institutional priorities:

We don't have the resources that we need for our patients. Like we don't have good staffing on labor and delivery because the hospital won't pay nurses enough… We'll run out of supplies that are like really basic sometimes and you wonder how does a hospital run out of this? So, it's constantly like, trying to do your best for your patients with the limited time and the limited resources that you have. (P09, MD, Muslim)

**10b. Malignant work culture.** At the level of the institution, disconnected administration and malignant work culture were named:

I think we are seeing a lot more physician burnout, healthcare burnout from just excessive hours, and probably a little bit of our own work culture being a little malignant. So, if you make a mistake, you're kind of ostracized. We have these just bizarre expectations that all physicians should never make a mistake. All nurses should never make a mistake. So, I always use the analogy to my nurses, because they love to pick on when you make a mistake. And I always say like, well, what about a quarterback? Does a quarterback always throw a touchdown pass perfectly? No. Does a pitcher always throw a perfect game? No. So and then I feel like adding on 24-hour shifts and fatigue, you're obviously going to create more mistakes, and professional athletes aren't expected to do that. So why are we? Pilots are expected to sleep well, why are we not? (P10, MD, Christian)

The threat of lawsuits and the tendency of infighting by providers seeking to place blame in the case of an adverse outcome were another barrier identified related to organizational culture.

It is noteworthy in the context of this study that some participants observed an increasing prevalence of mindfulness practices in medical training with skepticism. “I don't know if I totally buy into mindfulness" (P20, RN, Christian). Along these lines, another participant elaborated on the broader issues related to promoting a healthy work environment:

When we're talking about burnout, and wellness and all of these things. There is so much, sometimes like a lip service, it's not like you could truly say, let me take this, all this time to go have this spiritual connection. (P19, MD, spiritual not religious)

**10c. Societal issues.** Economic and political factors driving institutional policies were cited often with a plain recognition of "a broken system" (P08, PA, Christian) of healthcare. Aspects of this subtheme include insurance company policies, the social determinants of health and broader processes that form the basis of health disparities, and the increasing patient load related to poor public health as well as limited access to care facilities. For example, one participant said, “We went up 1,000 deliveries this year. We went from 2,000 to 3,000 deliveries in a year. That's a lot of pregnant people” (P08, PA, Christian). Considering the magnitude of social issues, one provider expressed the following:

I think a lot of folks who care about a lot of these kinds of issues, you don't feel like you're ever doing enough. … amongst all the things I'm trying to accomplish right now, like, how do I fit that in? … I find myself at work, I get very distressed when I take a quick glance around the room and realize my Black patient, the only person they can relate to is the person who brought them their meal, or the person who's cleaning… what does that say to these people? And of course, that's decades of work …and education that has to change. So, it's like this big, insurmountable thing. (P04, MD, not spiritual nor religious)

The perspectives that providers shared on the facilitators and barriers to supporting their spiritual well-being reflect a multilevel perspective. The themes identified here emphasize the individual and healthcare organizational levels. Facilitators at the individual level include provider skills and behaviors at work, self-care out of work, and personal attributes. Facilitators at the organizational level include structural and cultural support. With respect to barriers, burnout and isolation exist at the individual level, and competing institutional priorities, malignant work culture, and societal issues were identified at the organizational level. These facilitators and barriers were described by participants in relationship to their perception of their own spiritual well-being as maternal healthcare providers and are consistent with the definition of spirituality used to guide this study.

## Discussion

Understanding the views and attitudes of providers toward spirituality in maternal healthcare is a first step toward planning and implementing programs to support providers in offering spiritual care, which has the potential to contribute to improving the overall quality of maternal healthcare. Provider perspectives contributed potential key elements to include in spiritual care training programs. These included defining spirituality, the relationship of provider spiritual well-being to spiritual care for patients, and the facilitators and barriers to provider spiritual well-being in maternal healthcare.

Definitions of spirituality were commonly related to ideas of meaning and purpose, connectedness, and tied to direct personal experience, reminiscent of findings from previous work (Considine, 2007; de Diego-Cordero et al., 2023; Rogers et al., 2020; Selby et al., 2017). Despite differing ways of defining spirituality, all the participants recognized and valued the positive impact of spirituality on health and the positive aspects of spirituality to cope with illness, as in prior research (de Diego-Cordero et al., 2023). There was a prevalent association with religion, but also a sense that spirituality is a broader concept. Rogers and colleagues also noted confusion among a sample of nurse practitioners in differentiating between spirituality and religion (Rogers et al., 2020).

This study contributes the perspective that “spirituality is about connection” (Theme 3), which allows patients to bring their own understanding of spirituality, including or excluding any religious connotations, to the encounter in an unproblematic way. Similarly, Rogers et al. (2020) suggest that providers recognize that spirituality is connected to hope, meaning, and purpose to operationalize spirituality in “spiritually competent practice” (Rogers et al., 2020, p. 15). They offer the dual construct of availability and vulnerability as a frame to guide secular practitioners in their efforts to provide spiritual care. Simplifying the conceptual framework of spirituality and spiritual care in these ways may empower providers to navigate perceived difficulties in spiritual care, such as feeling rushed (Theme 8a. Individual-level barriers: Burnout; Best et al., 2016) or navigating different beliefs of patients (Theme 5. Spiritual care involves navigating different beliefs of patients and providers; Considine, 2007).

This approach aligns with the efforts of Selby et al. (2017) to operationalize spiritual care through guiding providers into a heightened state of being with patients rather than an action-based, checklist approach to spiritual care. Selby and colleagues compared the perspectives of providers and patients with respect to spirituality and spiritual care and found that providers often adopted a “task-oriented” approach to spiritual care that risks missing the cues of patients to engage on the topic of spirituality (Selby et al., 2017, p.2017, p. 143). Both patients and providers indicated the importance of listening, “being with,” and social support (Selby et al., 2017, p. 145). Similarly, Best et al. (2016) found that providers reported more experienced physicians listened to patients and waited for cues that they were ready to discuss spiritual issues (Best et al., 2016). These findings highlight the need for a broad understanding of spirituality and the importance of provider spiritual well-being as a basis for providing spiritual care to patients. In this study, Theme 4 (Provider spiritual well-being is favorable for providing spiritual care) speaks to the importance of spiritual well-being of providers and complements previous research exploring this relationship to providing spiritual care (Best et al., 2016; Jones et al., 2021; Paal et al., 2015).

This study adds to the research on provider well-being by contributing to the area of spiritual well-being, specifically addressing a call to expand the approaches to addressing the prevalent issue of provider burnout (Collier et al., 2021). Collier and colleagues encourage a fuller discussion of religion and spirituality among healthcare providers to progress toward a holistic biopsychosocial–spiritual model of healthcare. Supporting providers’ spiritual well-being may be an effective strategy in cultivating resilience to the occupational stressors of the medical environment (de Diego-Cordero et al., 2022). For instance, Harris & Tao (2022) found spiritual well-being to be inversely associated with exhaustion and depersonalization, two aspects of occupational burnout.

Providing education and opportunities to practice spirituality is one way to directly improve outcomes of providers’ spiritual well-being. The Mantram Repetition Program (MRP) developed by Jill Bormann and colleagues through work with the Veterans Administration is an example of an evidence-based program that is effective in improving healthcare providers’ spiritual well-being (Bormann et al., 2017; Oman et al., 2020). Research indicated that providers improved in measures of spiritual well-being and mindfulness after participating in the six 50-min classes, which were delivered online every two weeks over a period of 3 months. Results were sustained at a 3-month post-intervention follow-up. In addition to demonstrating effectiveness, the program appears to meet implementation criteria for healthcare professionals and is sustainable—72% of participants reported continued practice of all program tools at follow-up (Bormann et al., 2017).

The barriers to provider spiritual well-being identified in this study were consistent with those of previous research and primarily related to burnout and structural constraints. Burnout due to heavy workloads and a high-stress environment are the most cited forces that undermine provider well-being in the literature (e.g., National Academies of Sciences, 2018). Providers suffer from a lack of time for personal care and nurturing social and familial relationships, contributing to feelings of isolation. Providers also experience a level of distress in their recognition that patients do not receive the holistic care that they would like to provide due to limits on time available to meet with each patient. Ripp and colleagues describe these pressures as contributing to “moral fatigue” of providers, in which providers recognize that the goals of the systems in place often seem to be at odds with what is in the best interest of the patient (Ripp et al., 2017, p. 914).

Although structural barriers to provider well-being are driven by larger social, economic, and political forces, certain organizational-level barriers are within the sphere of influence for individual providers. These include the cultural norms that providers hold themselves to, such as tendencies toward perfectionism under high stress and stigmatizing mental health counseling. These findings reinforce the idea of spiritual well-being as an indispensable and interconnected dimension of overall well-being (Oliver et al., 2018). For example, Theme 7a (Individual-level facilitators: skills and behaviors in the clinic) describes elements of occupational well-being and good clinical practice (e.g., being prepared for clinical appointments, not taking patient rudeness personally, etc.) in the context of supporting providers’ spiritual well-being.

Other organizational barriers require support from institutional administration to prioritize provider spiritual well-being by providing adequate resources and compensation, not only in pay, but in adequate benefits and time off. The intersection of organizational aspects with individual impacts is a common theme in the literature on provider well-being (e.g., Sinskey et al., 2022; Tawfik et al., 2019). It is unsurprising, then, that proposed solutions have been addressed at system level changes (e.g., Hamric & Epstein, 2017). The findings of this study support the basis for these efforts to support provider well-being as a foundation for spiritual care.

### Future Directions

Although provider spiritual well-being is an essential element for spiritual care of patients, it is not necessary prior to engaging with spiritual care training programs. In fact, studies have shown that healthcare professionals in spiritual care training programs reported benefits to their own sense of spirituality (Jones et al., 2021; Paal et al., 2015). Provider spiritual health, spiritual well-being, and spiritual integrity all benefited by participation in spiritual care training. Providers reported that increased awareness of spiritual care strategies allowed them to apply these strategies to their own lives and emphasize spiritual growth to a greater extent (Jones et al., 2021). While spiritual care training programs can support spiritual well-being of providers, it is not clear that the benefits from these programs alone are sustainable. It is recommended that such training occurs in conjunction with addressing the barriers to provider spiritual well-being described in this paper (Theme 10. Organizational-level barriers), and not as a substitute for transforming the relevant contextual and organizational factors.

By delving into the layered meanings of spirituality from the perspective of maternal healthcare professionals, the findings of this study are applicable to developing interventions to improve the spiritual well-being of providers and spiritual care of patients. Implications for practice include identifying strategies that may be effective in promoting acceptability of spiritual care interventions among providers, such as focusing on the benefits to maternal health outcomes, opportunities to improve communication and trust in the patient–provider relationship, and the intersection with provider spiritual well-being. Organizational and provider concerns related to time available with patients, fear of offending patients or overstepping professional bounds, and overcoming a skepticism bordering on cynicism of the genuineness of organizational efforts to support provider well-being must all be tactfully addressed in developing training materials.

Implementing provider peer groups to discuss the spiritual aspect of maternal healthcare for providers and patients is recommended as a component for spiritual care training. This was a suggestion that came directly from a participant and is a way of leveraging one of the facilitators identified (Theme 7a. Individual-level facilitators: skills and behaviors in the clinic) through the opportunity for peer-to-peer discussion and mentoring. Bringing providers together can also help to address the need for cultural growth at the organizational level by assuaging fears of appearing unprofessional to colleagues. Discussion groups may also contribute to provider spiritual well-being by combatting feelings of isolation identified in this study.

Further steps to promote provider spiritual well-being include offering additional forms of spiritual support, such as evidence-based programs like the MRP. The findings of this study are helpful to understand the needs and priorities of healthcare professionals with respect to how best to implement programs like the MRP. For example, a focus on skill development and creating an organizational culture that values holistic well-being, two features of the MRP, aligns with facilitating factors at the individual and organizational levels that were identified in the findings. This alignment indicates a potential fit of the MRP for the organizations represented by the maternal healthcare providers interviewed in this study.

### Limitations

While this study has several strengths, such as the qualitative approach to explore a timely and important topic, and the use of peer debriefers and member checking of the findings to enhance the rigor and trustworthiness of the study, there are also a few limitations to note. Related to the sampling approach, the findings of this study may be bound by limited access to the provider population. The rates of burnout are notoriously high for healthcare workers. The interviewer (TN) lacked an insider’s experience with the participant population, having had no medical training or healthcare worker experience. This may have limited rapport with the participant, particularly related to medical and administrative aspects of working as a maternal healthcare provider.

The findings are limited to the self-reported experiences of the participants, which may miss additional data relevant to the research questions, such as evaluating the experience of patients. Finally, the nature of this qualitative study, and its aim at ideographic generalization, does not provide a sense of the prevalence of the perspectives of maternal healthcare providers toward spiritual care. Although the findings are likely to represent the types of issues related to this topic, they are unable to convey the magnitude of these issues, which is necessary to consider when making decisions related to allocation of scarce resources and should be studied further with quantitative and mixed-method approaches.

## Conclusion

The findings of this study both inform the design and implementation of spiritual care within maternal healthcare and point to essential areas for further research on spiritual care. The integration of spiritual care into maternal healthcare is a promising approach to improving the quality of maternal healthcare as a contribution to the broad efforts to improve both healthcare provider health and maternal health outcomes. The perspectives of providers and the role of provider spiritual well-being are central to the continued development of this field. This study presents opportunities to address provider needs in implementing spiritual care by attending to the spiritual well-being of providers themselves.

## Appendix 1: Interview Guide

The information on this page is for my reference and is not intended to be shared directly with the participants. The definitions may be shared if needed to advance the discussion with the participant. The following script and questions were used to guide 60–90-min interviews.

### Research Questions

1. How do providers define spirituality and mindfulness and their role in patient care?
2. How do providers perceive their own spiritual well-being as a strategy to address the spiritual needs of patients in maternal care?
3. What are the perceived barriers and facilitators to achieving personal spiritual well-being and integrating spiritual care into maternal care?

### Definitions

**Spirituality:** “the aspect of humanity that refers to the way individuals seek and express meaning and purpose and the way they experience their connectedness to the moment, to self, to others, to nature, and to the significant or sacred” (Puchalski et al., 2009, p. 887).

**Spiritual care:** person-centered care that attends to a patient’s sense of meaning, hope, and inner strength (Kelly, 2012). Spiritual care values wholeness and healing over treating a specific condition or disease and “serving the whole person – the physical, emotional, social, and spiritual” (Puchalski, 2001, p. 352).

**Mindfulness:** “paying attention…on purpose, in the present moment, and nonjudgmentally” (Kabat-Zinn, 1994, p. 4).

## Introduction

Thank you for taking the time to meet with me today and for your valuable contribution to this research. The goal of this study is to learn from providers to better understand the role of spirituality, spiritual well-being, and spiritual care in maternal healthcare and how provider spiritual well-being may help improve maternal health outcomes and equity.

My interest in this work is motivated by a desire to improve the model of maternal healthcare, especially for Black women, who experience the worst rates of maternal morbidities, mortalities, and infant mortalities. I recognize that there are multiple layers of social identity that I experience, including my being a White, cisgender, heterosexual male, among others, that influence and limit my perception of the issue of maternal health disparities. Personally, my approach is informed by my experience as the husband of a Black woman and our shared experiences of maternal healthcare during two pregnancies and by my personal understanding and practice of spirituality. I am a PhD candidate in the Family, Youth, and Community Sciences Department. My training is in social science. I don’t have any medical training or background working in healthcare.

A major focus of this interview is on your personal experiences of spirituality. This may be very deep, personal, and vulnerable, and I want you to feel safe sharing your experiences and perspectives. We can take our time and even take a pause at any point if you would like to. Every effort will be made to de-identify your responses and to protect your anonymity. You will have the opportunity to review the interview transcript for accuracy and to provide comments on any report produced prior to submission for publication. I am deeply grateful for your willingness to share your personal experiences and insights.

### Questions

#### Opening

- Do you have any questions for me before we begin? Please feel free to ask me questions at any point during the interview.
- Please tell me about your role as a maternal healthcare provider. What do you do?

### RQ1: How Do Providers Define Spirituality and Mindfulness and Their Role in Patient Care?

- People have many ways of understanding spirituality. What does spirituality mean to you? Can you give me an example of an experience that helped you understand what spirituality means to you?
- Do you relate spirituality to religion? If so, how?
- Do you relate spirituality to mindfulness? If so, how?
- Was there ever a time when you felt that your sense of spirituality contributed to your career choice as a maternal healthcare provider? Can you tell me about that?
- Can you tell me about a time when you attended to a patient’s spiritual needs?

### RQ2: How Do Providers Perceive Their Own Spiritual Well-Being as a Strategy to Address the Spiritual Needs of Patients Who Identify as Black Women in Maternal Care?

- How do you take care of your own spiritual well-being before, during, and after patient encounters? Can you walk me through it?
- Can you describe a time when your sense of spirituality was enhanced by your role as a maternal healthcare provider?
- What about the opposite? Can you describe any time when you experienced a disconnect between your spirituality and your professional role?
- Black women are a group who experience higher rates of maternal mortality. In some cases, a need for a greater level of spiritual care in maternal healthcare has been indicated by Black women.
- How does understanding the maternal health disparities faced by populations of Black women impact your spiritual well-being?
- Given the benefits of racial and cultural concordance in care provision and the lack of representation of Black women in medicine, how might providers’ spiritual well-being influence the quality of care for Black women?
- Can you think of any instances when your spirituality (or perhaps that of a colleague) contributed to providing healthcare to a patient who was a Black woman?
- **Clarifying (contrast) question:** (It sounds like your… OR… I imagine) spirituality and spiritual well-being as a provider (is / may be) something that’s personal and private, but also can be an asset in serving your patients. Can you tell me more about when it is appropriate to draw on your spirituality in patient care and when it is inappropriate?

### RQ3: What Are the Perceived Barriers and Facilitators to Achieving Personal Spiritual Well-Being and Integrating Spiritual Care into Maternal Care?

- Where do you see opportunities to support the spiritual well-being of maternal care providers in the delivery of your medical care services?
- Who do you think might be champions to support these opportunities?
- What are some of the challenges to your experience of spiritual well-being in your professional role?
- You mentioned [challenge, e.g., time pressure]. How do you talk to your colleagues about this issue?

### Closing

- Is there something that you might not have thought about before that occurred to you during this interview related to spirituality and spiritual well-being for maternal healthcare providers?
- Thank you for taking the time to speak with me today! Your responses are greatly appreciated and as a token of our gratitude, we will send you your $50 Amazon e-gift card code to the email address that you provided. May I follow up with you for recommendations of providers who may be willing to participate in this study?
